# Exercise-Based Rehabilitation Delivery Models in Comorbid Chronic Pulmonary Disease and Chronic Heart Failure

**DOI:** 10.3389/fcvm.2021.729073

**Published:** 2021-10-13

**Authors:** Audrey Borghi-Silva, Adriana S. Garcia-Araújo, Eliane Winkermann, Flavia R. Caruso, Daniela Bassi-Dibai, Cássia da Luz Goulart, Snehil Dixit, Guilherme Dionir Back, Renata G. Mendes

**Affiliations:** ^1^Cardiopulmonary Physiotherapy Laboratory, Physiotherapy Department, Federal University of Sao Carlos, Sao Carlos, Brazil; ^2^Graduate Program in Comprehensive Health Care, Universidade de Cruz Alta/Universidade Regional do Noroeste do Estado do Rio Grande do Sul, Ijuí, Brazil; ^3^Postgraduate Program in Management and Health Services, Ceuma University, São Luís, Brazil; ^4^Department of Medical Rehabilitation Sciences, College of Applied Medical Sciences, King Khalid University, Abha, Saudi Arabia

**Keywords:** chronic heart failure, chronic obstructive pulmonary disease, rehabilitation, exercise, comorbidities, non-invasive ventilation, aging

## Abstract

Among the most prevalent multimorbidities that accompany the aging process, chronic obstructive pulmonary disease (COPD) and chronic heart failure (CHF) stand out, representing the main causes of hospital admissions in the world. The prevalence of COPD coexistence in patients with CHF is higher than in control subjects, given the common risk factors associated with a complex process of chronic diseases developing in the aging process. COPD-CHF coexistence confers a marked negative impact on mechanical-ventilatory, cardiocirculatory, autonomic, gas exchange, muscular, ventilatory, and cerebral blood flow, further impairing the reduced exercise capacity and health status of either condition alone. In this context, integrated approach to the cardiopulmonary based on pharmacological optimization and non-pharmacological treatment (i.e., exercise-based cardiopulmonary and metabolic rehabilitation) can be emphatically encouraged by health professionals as they are safe and well-tolerated, reducing hospital readmissions, morbidity, and mortality. This review aims to explore aerobic exercise, the cornerstone of cardiopulmonary and metabolic rehabilitation, resistance and inspiratory muscle training and exercise-based rehabilitation delivery models in patients with COPD-CHF multimorbidities across the continuum of the disease. In addition, the review address the importance of adjuncts to enhance exercise capacity in these patients, which may be used to optimize the gains obtained in these programs.

## Highlighted

- Patients with coexistence of COPD-CHF present a further impaired exercise capacity and health status associated to mechanical-ventilatory, cardiocirculatory, autonomic, gas exchange, muscular, ventilatory, and cerebral blood flow disfunctions.
- COPD-CHF calls for a multifaceted and integrated disease management based on pharmacological and non-pharmacological treatment focused in an exercise-based cardiopulmonary rehabilitation delivered emphatically by professionals aware of multimorbidity care. Adjuncts as non-invasive ventilation, may enhance the exercise tolerability, performance, and consequently better physiological adaptations and outcomes.
- Further RCT studies are necessary to investigate randomized clinical trials to investigate the exercise-based rehabilitation in COPD-CHF overlap in a more systematically way in terms of criteria of inclusion, types of CHF (preserved or reduced), components, and outcomes.

## Introduction

Chronic obstructive pulmonary disease (COPD) and chronic heart failure (CHF) are prevalent noncommunicable chronic conditions (1). Aging is a diverse and complex process related to chronic diseases developing, attributed in parts to the long exposure to an unhealthy lifestyle. This process favor the augment in the prevalence of mutually coexistence of diseases (2), as the COPD-CHF that has been highlighted in recent years and associated with poor adverse outcomes (3, 4).

COPD is characterized by chronic obstruction and progressive limitation to airflow, which is not fully reversible (5). On the other hand, CHF is a complex clinical syndrome in which the heart is unable to maintain tissue perfusion according to metabolic demands (6). CHF is defined according to left ventricular ejection fraction (EF) and although both types are possible to coexist, the reduced EF (HFrEF) is the most cited criterion to diagnose HF in patients with COPD.

Both COPD and CHF share the systemic inflammatory process and increased oxidative stress as a pathophysiological basis and smoking is the main etiology involved (7). This association imposes systemic consequences and impact on the quality of life, morbidity and mortality (3, 4). Systemic consequences include changes in cardiopulmonary and autonomic function, peripheral and respiratory muscle weakness (8) and can magnify the symptoms, leading to a marked negative impact on mechanical-ventilatory, cardiocirculatory, autonomic, gas exchange, muscular, ventilatory, and cerebral blood flow, further impairing the reduced exercise capacity and health status than either condition alone (9).

In the face of this complexity, the biggest challenge in the clinical practice involving patients with COPD-CHF is the establishment of an integrated and individualized approach focusing among other things on the management of dyspnea (3), exercise intolerance (10), functionality aimed to improve the survival and quality of life.

## Impact of COPD-CHF on Exercise Capacity and Symptoms

Exercise intolerance may be considered multifactorial in COPD-CHF (11). Pathophysiological process of both diseases are related to imbalances of oxygen transport, cardiovascular, respiratory, muscle, and brain disorders (12). In this context, there are several lines of evidence suggesting that oxygen delivery to working muscles may be critically impaired during dynamic exercise in COPD-CHF patients (9). In addition, another impact in patients with COPD-CHF are ventilatory inefficiency and peripheral muscle dysfunction characterized by reduced muscle strength, promoting negative consequences on exercise performance (9, 13).

Studies have demonstrated that reduced oxygen supply (O_2_), systemic inflammation and increased oxidative stress can contribute to respiratory muscle dysfunction (9, 12–14). In this context, an understanding of the interaction between the mechanisms that lead to symptoms during exercise such as dyspnea, can provide useful information about both diseases (15). Exercise intolerance may be due to critical mechanical-ventilatory restrictions or hypoxemia, demonstrating the contribution of COPD disease (16), or even exercise may be interrupted due to complaints of discomfort in the lower limbs, demonstrating the contribution of both diseases (9).

**Figure 1:**
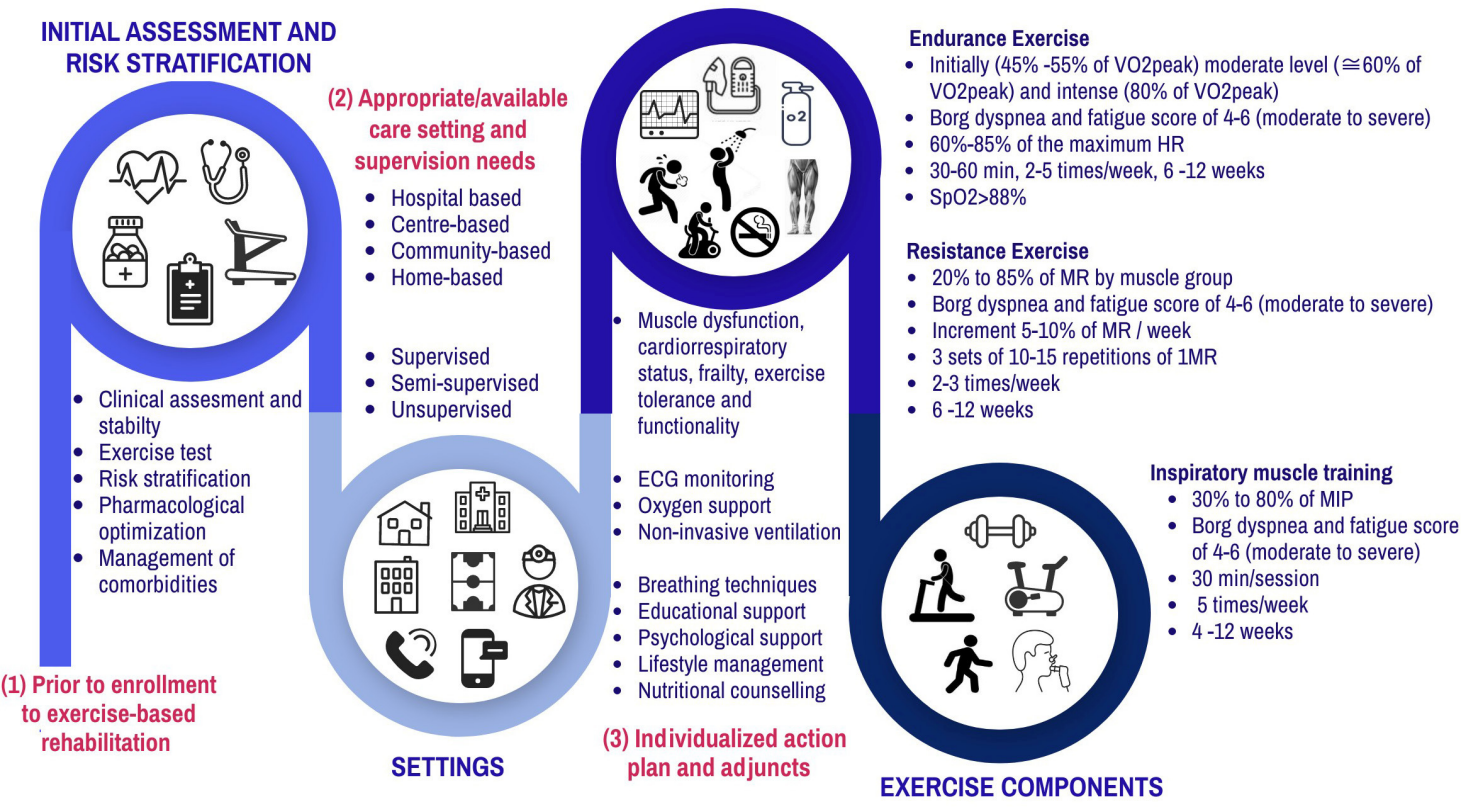
Summary of components of Exercise-based rehabilitation on the population with COPD-CHF or COPD-Cardiovascular comorbidities. *V*O_2peak_, oxygen consumption; HR, heart rate; MR, maximum repetition; MIP, maximum inspiratory pressure; SpO_2_, Peripheral oxygen saturation.

Cardiopulmonary rehabilitation (CR) for COPD and CHF patients consists of a program of structured, individualized, and supervised physical exercises, usually performed for a period of 6–12 weeks (17). Furthermore, CR in general is interdisciplinary, including health education measures, and its primary objective is to improve exercise capacity and, consequently, dyspnea (18), as well as preventing secondary cardiac events (19). CR may reduce the risk of hospital admissions for all causes, as well as specific hospital admissions for CHF in the short term (up to 12 months). Additionally, CR attributes an important improvement in the quality of life of these individuals (20). Prior to enrollment to exercise rehabilitation, patients should be clinically assessed, an exercise test and risk stratification is recommended. Patients should present clinical stability with no contraindications to exercise training (such as unstable angina, respiratory failure, and severe electrolyte imbalance) and should have optimized comorbidities and pharmacological treatment. Moreover, the risk stratification and need for electrocardiogram monitoring, oxygen supplementation and supervision level must be assessed and programmed individually. During the CR sessions, it is important to remain aware of signs regarding decompensating, such as disproportional increased shortness of breath and fatigue, increased body weight and edema, worsening of pulmonary auscultation, increased coughing, and intolerance to supine position. Breathing techniques, educational support, psychological support, lifestyle management and nutritional counseling are allied components. Desveaux et al. (21) also concluded that motivation and incentive, as well as access to appropriate facilities, are key resources to support adherence to the prescribed activity. Thus, the multifaceted approach achieved by the integration between physical training, pharmacological, and nutritional care in a personalized way (21) is warranted to these patients who present the coexistence of COPD-CHF. Figure 1 summarizes the recommendations and components of an integrated CR.

A recent review conducted by Anderson et al. (22) highlights that home-based rehabilitation seem to be equally effective than those performed in CR centers when considering improving the quality of life in patients post myocardial infarction or revascularization and heart failure. In addition, it supports the continued expansion of home-based and evidence-based CR programs. Moreover, according to Anderson et al. (22) participating in a more traditional program with supervision carried out at a center or in a home program reflects the local availability, as well as the individual preference of the patient.

## Exercise-Based Rehabilitation Delivery Models for COPD-CHF Patients

Although there is strong evidence supporting exercise-based rehabilitation as standard of care for people with COPD or CHF, there are only few RCTs demonstrating the models and effects of rehabilitation specifically for mutually combined COPD-CHF diagnosis. In most of the studies included in the review we found that studies primarily focused on the COPD population whereas cardiovascular impairments had a secondary emphasis.

Regardless of basic cardiac or pulmonary rehabilitation approaches, COPD and CHF have similar systemic manifestations such as limitation in exercise capacity, skeletal muscle dysfunction, dyspnea, deconditioning, reduced level of activities of daily living, and quality of life (23). To the best of our knowledge until now, there are only two studies focusing on rehabilitation specifically in patients with coexisting COPD-CHF (24, 25). Smyrnova et al. (23) carried out hospital based yogic breathing as an adjuvant therapy in PR to enhance the effects on the intervention group and found a significant difference compared with the control group. Bernocchi et al. (24) carried out a telerehabilitation home-based program with an individually tailored exercise program for each participant who were primarily having COPD-CHF diagnosis and found that home-based intervention was feasible and effective in improving exercise capacity. Most of the included studies which were performed with interventions in rehabilitation centers found improvements in objective measures such as exercise capacity, muscle strength, and subjective measures such as a sensation of dyspnea and quality of (QoL) assessed by Minnesota and saint George. Whereas, the home-based program with regular follow-up and counseling also demonstrated similar improvements, Table 1 provides information on rehabilitation exercise-based delivered models and their effects on patients with coexisting COPD-CHF (24, 25) or COPD-cardiovascular comorbidities (26–32). The main approach to rehabilitation delivery was undertaken in an outpatient setting, center-based or/and home-based, and almost all programs were comprehensive including exercises, education, and lifestyle change support. The program provided both supervised or unmonitored sessions and the exercise training was usually offered in an individually tailored manner.

**Table 1:** Exercise-base rehabilitation models and their effects on the population with COPD-CHF or COPD-Cardiovascular comorbidities.

**References**	**Study design**	**Sample size**	**Participants and average age**	**Setting**	**Type of care**	**Interventions**	**Components**	**Effect of intervention**	Risk of bias*
Smyrnova et al. (23)	Unclear	*n* = 102	COPD+CHF Average age: CG:67 ± 6 IG: 69 ± 6 years CHF type: not reported Average EF: not reported	Inpatient	Hospital based	CG: SC IG: SC + full yogic breathing	CG: PR program IG: PR and yogic breathing	Full yogic breathing + SC is associated with more pronounced ↑ exercise tolerance (6MWD), ↓ dyspnea (Borg scale), ↓ length of hospital stay, ↓ functional class	4
Bernocchi et al. (24)	RCT	*n* = 112	COPD+CHF Average age: IG: 71 ± 9 CG: 70 ± 9 years CHF type: not reported. Average EF: IG: 44.5 ± 12.4% CG: 43.3 ± 13.2%	Outpatient	Home based	CG: SC IG: tele rehabilitation educational intervention + personalized exercise program	CG: Educational program, medications and oxygen IG: Aerobic and resistance exercises + reinforcement on lifestyle changes	Rehabilitation was feasible and effective in ↑exercise capacity (6MWD), ↑ physical activity profile (PASE), ↑QoL (MLHFQ and CAT) and ↓disability (Barthel), ↓ dyspnea (MRC) and ↓time to hospitalisation/death	5
Berry et al. (25)	RCT	*n* = 140	COPD + HD comorbid (*n* = 51) Average age: 1st group = 67, 2nd group = 68 years HF type: not reported Average EF: not reported	Outpatient	Centre/Home based or community based	3 months supervised and then divided into: Short-Term (unmonitored) Or Long-Term Care (Supervised)	Aerobic exercise and upper-extremity resistance exercise training	Long term supervised care is more beneficial in ↑exercise capacity (6MWD), ↓disability (self-reported), ↓time to climb steps and overhead task	5
Berry et al. (26)	RCT	*n* = 176	COPD + HD comorbid (*n* = 79) Average age: TET: 66 ± 10/LAP: 66 ± 10 years HF type: not reported Average EF: not reported	Outpatient	Centre based	TET: Traditional exercises program or LAP: Behavioral Lifestyle program	TET: walking exercises + warm-up and cool down, LAP: education and self-reliance of exercise	Both were effective in increasing and maintaining moderate levels of physical activity (moderate physical activity—kcal/week)	6
Coultas et al. (27)	RCT	*n* = 325	COPD+CV comorbid (*n* = 198) Average age: 70 ± 9 years HF type: not reported Average EF: not reported	Outpatient	Centre based/Home based	SC or Moderate intensity lifestyle physical activity with a structured workbook	SC or Group workbook: telephone health coaching tailored to each participant	Home-based coaching intervention may ↓sedentary behavior and ↑physical activity levels (RAPA questionnaire), ↓rate of previous lung-related health care utilization	5
Charikiopoulou et al. (28)	RCT	*n* = 32	COPD+CV comorbid (*n* = 22) Average age: G1:64 ± 5 G2:67 ± 6 years HF type: not reported Average EF: not reported	Outpatient	Centre based	Individualized rehabilitation program Two groups, according to comorbidities	Exercises: aerobic, resistance and respiratory muscle training; breathing retraining, education, diet, and psychological support	PR seems to be beneficial for all patients in ↓dyspnea (MRC), ↑QoL (SGRQ and CAT), ↑exercise capacity (6MWD), independently of the presence, the number or the nature of their comorbidities	3
Lenferink et al. (29)	RCT	*n* = 201	COPD+HD comorbid SM: 69 ± 9 years UC: 68 ± 9 years HF type: not reported Average EF: not reported	Outpatient	Centre based/Home based	Patient-tailored self-management intervention (Individual and group sessions + phone call) or SC (group session and phone call)	SM: Educational program (disease, self-treatment, importance of physical fitness and exercise, diet and lifestyles behaviors, exacerbation actions) or SC: Educational symptom level and diaries	Patient-tailored action plans for COPD patients with comorbidities, ↓duration per COPD exacerbation and ↓risk of respiratory-related hospitalization during follow-up	7
Foy et al. (30)	RCT	*n* = 140	COPD + HD comorbid (*n* = 51) Groups average age: G1:67 ± 6 G2:68 ± 6 years HF type: not reported Average EF: not reported	Outpatient	Centre/Home based	3 months supervised and then divided into: Short Term (unmonitored) Or Long-Term Care (Supervised)	Walking and strength training. Short term therapy mainly home based, long term structured regime	↑ CRQ after 3 months and ↓dyspnea, fatigue, emotional function and mastery after long term Men derive significant benefits from extended training	4
Vasilopoulou et al. (31)	RCT	*n* = 150	COPD + CV comorbid (*n* = 42) Average age: GA:67 ± 10 GB:67 ± 7 GC:64 ± 8 years HF type: not reported Average EF: not reported	Outpatient	Centre/Home based	Home-based maintenance tele rehabilitation in an individualized action plan Hospital-based: Outpatient maintenance rehabilitation or SC treatment	Home-based: exercise, psychological support; dietary and SM advice Hospital-based: exercise, physiotherapy, dietary and psychological advice SC: vaccination pharmacotherapy, oxygen	Home-based maintenance tele-rehabilitation is equally effective as hospital-based outpatient in ↓ risk for acute COPD exacerbation and hospitalizations and; in preserving exercise capacity (6MWD), peak work rate, QoL (dyspnea and mMRC)	5

CHF, chronic heart failure; COPD, chronic obstructive pulmonary disease; CAT, COPD Assessment Test; CV, cardiovascular; EF, Ejection fraction; HD, heart disease; CG, Control Group; IG, Interventional Group; SC, standard care; MLHFQ, Minnesota Living with Heart Failure; MRC, Medical Research Council; QoL, Quality of life; PASE, physical activity profile; PR, Pulmonary rehabilitation; 6MWD, six-minute walking distance; TET, Traditional exercises program; LAP, Behavioral Lifestyle program; CRQ, Chronic Disease Respiratory Questionnaire; SM, self-management; RAPA, Rapid Assessment of Physical Activity. ^*^Risk of Bias was assessed by PEDro Scale Tool.

### Exercise Training Programs in Patients With COPD-CHF

Although statements have suggested exercise-based rehabilitation as a standard of care for people with COPD or CHF, there are few RCT studies that demonstrate the models provided specifically for the COPD-CHF overlap syndrome. A specific analysis of exercise-based rehabilitation, in both isolated conditions, shows a similarity in the specific type of interventions and training prescription, such as aerobic, resistance, and inspiratory muscle training. We carried out an analysis of the articles mentioned in Table 1, in a total of nine studies covered, we selected five RCTs that presented a better methodological description of the applied rehabilitation protocols (24, 25, 30–32). Only two studies (23, 24) evaluated the effect of rehabilitation on the coexistence of diseases. Table 2 presents a summary of the three main types of exercise adopted, with intensity, duration, frequency, and supervision according to current evidence on an exercise-based rehabilitation program. In addition, the analysis described in the following paragraphs highlights the main forms of exercise prescription in this population and, at the same time, demonstrates the similarity between them in these three types of exercise-based therapy (Table 2).

**Table 2:** Summary of exercise components and prescription (intensity, duration, frequency, and supervision) according to current evidence of exercise-based rehabilitation program to COPD-CHF or COPD cardiovascular comorbidities.

**Exercise**	**Intensity**	**Duration**	**Frequency and duration**	**Supervision/control**	**Safety**	**Care to be taken**
Aerobic exercise (24, 25, 30)	Initially (45–55% of *V*O_2peak_) moderate level (?60% of *V*O_2peak_) and intense (80% of *V*O_2peak_) 60–85% of the maximum HR Borg dyspnea score of 4–6 (moderate to severe)	30–60 min	2–5 times a week 6–12 weeks	Borg dyspnea and fatigue score of 4–6 (moderate to severe), SpO_2_ > 88% and HR should be below the maximum HR predicted by age	Exercise-based rehabilitation is safe, and it has no hemodynamic, respiratory, and/or musculoskeletal complications during the execution of exercise protocols (23, 24)	Patient education to perform the exercises and recognize the symptoms of dyspnea and fatigue (23, 24, 31)
Resistance Exercise (24, 25, 31)	From 20 to 85% of MR by muscle group Increment 5–10% of MR per week	3 sets of 10–15 repetitions of 1MR	2–3 times a week 6–24 weeks	Borg dyspnea and fatigue score of 4–6 (moderate to severe)		
Inspiratory muscle training (33)	30–80% of MIP	30 min	5 times a week 4–12 weeks.	Borg dyspnea and fatigue score of 4–6 (moderate to severe)		

*VO_2peak_, oxygen consumption; HR, heart rate; MR, maximum repetition; MIP, maximum inspiratory pressure; SpO_2_, Peripheral oxygen saturation*.

One of these modalities is aerobic training aiming to condition the muscles involved in walking and improving cardiorespiratory fitness (34). Exercises with an intensity >60% of the peak work rate in an incremental or constant test, performed for 30–60 min, are necessary to achieve these objectives (34). Interval training is proposed as an alternative to continuous training, especially for individuals who are unable to tolerate continuous high-intensity aerobic training due to intolerable symptoms and, therefore, shorter sessions should be advised in order to accumulate at least 30 min of exercise. Aerobic training per session (35). The training intensity can also be defined and/ or titrated according to Borg's dyspnea scores (4–6, moderate to intense) (32). A frequency of 3–5 sessions per week is recommended (35). Walking and the cycle ergometer are considered the best training modalities (32).

Aerobic exercise aims to minimize limiting symptoms such as skeletal and respiratory muscle dysfunction, dyspnea, and fatigue in these patients. These symptoms reduce patients' ability to exercise and compromise cardiac fitness, which further limits their exercise tolerance, creating a vicious downward spiral that can eventually lead to generalized debility and immobility. However, in some cases aerobic exercise is contraindicated. Patients with unstable heart and lung disease, locomotor difficulties that preclude patient exercise, significant cognitive, or psychiatric impairment that could lead to an inability to follow simple commands in a group setting should not be referred for rehabilitation (24, 25, 35).

Another modality is skeletal muscle resistance training (36). Evidence shows that resistance training alone or combined with aerobic training improves peak VO_2_, QoL, and walking performance in patients with isolated heart failure (37, 38). While recognizing that the ideal prescription for resistance training for people with COPD-CHF has not been determined, the statement refers to the British Thoracic Society guidelines for prescribing resistance exercises (35). Most evidence recommends a prescription based on the maximum repetition test (1MR) (36). The overload principle is emphasized, which involves increasing the dosage of exercise over time to maximize gains in muscle strength and endurance. This can occur by increasing the weight, increasing the number of repetitions per set, increasing the number of sets of each exercise and/or decreasing the rest period between sets or exercises (36).

The exercise modality that is also performed in both pathologies is inspiratory muscle strength training (IMT) (39). IMT tries to improve the strength and resistance of the respiratory muscles by means of devices that allow inhalation against resistance within a certain limit (39). IMT can reduce dyspnea by favorably altering the relationship between the current inspiratory pressure generated and the maximum inspiratory pressure (34) and reducing the impairment of dynamic hyperinflation (37). The prescription of this modality includes a minimum load ranging from 30 to 80% of MIP assessed by manovacuometry. Most studies include prescriptions that vary with daily training (5–7 times a week) for a considerable period (around 30 min daily), some studies emphasize the division of this time (2 sets of 15 min each), and some studies include the number of repetitions in each set (12–15 repetitions) in their protocols (39).

In the absence of a clear protocol to guide the practice, physical therapists should use clinical assessment and provide carefully monitored and supervised exercises and a multidisciplinary and collaborative team approach for training, prescription and progression of individualized exercises (35). An initial and continuous assessment that includes the severity of the disease and symptoms, the comorbidities and the patient's goals should be emphasized. This should be combined with individual and aggregate measurement and analysis of patient-centered results and exercise capacity (35). Finally, rehabilitation must emphasize sustainable exercise that translates into increased physical activity in the long term through safe and effective interventions.

The study provides evidence that it is safe and feasible to apply early (40) pulmonary rehabilitation in patients with acute exacerbation of COPD. Most studies do not report care needs during exercise sessions. The current evidence demonstrates that continued supervised maintenance exercise compared to usual care following pulmonary rehabilitation reduces health care use in COPD (41). Similarly in heart failure, the exercise training was associated with modest significant reductions for both all-cause mortality or hospitalization and cardiovascular mortality or heart failure hospitalization (42). Cardiac rehabilitation may improve all-cause mortality in the long term (>12 months follow up; RR = 0.88, 95% CI: 0.75–1.02). Moreover, the CR probably reduces overall hospital admissions in the short term (up to 1 year of follow-up and may reduce HF-specific hospitalization) (20).

### Adjuncts to Exercise Training Programs to Optimize Cardiac Rehab in COPD-CHF Patients

Several adjuncts associated with physical exercise have been applied in these patient populations, in their isolated or associated form in order to improve exercise capacity, reducing the overloaded respiratory muscles and consequently the breathlessness and increase muscle O_2_ availability. Noninvasive positive pressure ventilation (NiPPV) as an adjunct during exercise is able to acutely relieve the ventilatory muscles and improve the supply of peripheral O_2_ in these patients.

Previous studies have proven the positive effect of NiPPV on exercise tolerance, increase oxygen uptake and counterbalance mechanical ventilator changes in both COPD (43) and HF (44). A potential mechanism that could explain these benefits is the pulmonary mechanical effect that may improve cardiac function and regional vascular distribution (45, 46). In addition, recent evidence has demonstrated that NiPPV can also positively modulate heart rate variability during high-intensity exercise in COPD-CHF patients (47). The use of non-invasive ventilation during high intensity exercise (HIT) is thus recommended when the objective is to quickly emphasize peripheral muscle gains in a rehabilitation program when dyspnea symptoms can be the potential limiting factor for tasks that require greater efforts in these patients. Unfortunately, few studies have evaluated the effect of NiPPV during long-term rehabilitation programs in patients with COPD-CHF. Additionally, da Luz Goulart et al. (47) demonstrated that NiPPV applied through two pressure levels (Bilevel) 8–12 cmH_2_O to inspiratory pressure and 4–6 cmH_2_O for positive end-expiratory pressure produced a marked acute benefit on endothelial function associated with improved exercise tolerance during a bout of high-intensity aerobic exercise in patients with coexisting COPD-HF. The NiPPV improving peripheral oxygenation may also have influenced the local metabolic activity, causing an acute “restoration” of endothelial function in COPD-CHF patients (47).

Previously, Mazzuco et al. (48) have applied proportional assist ventilation mode of NiPPV (volume assist = 12–16 cmH_2_O = and flow volume assist = 4–8 cmH_2_O) and demonstrated an accelerated VO_2_ recovery kinetics following high-intensity exercise in COPD-CHF. Therefore, the NiPPV was proved to be an interesting adjunct in CR programs to sustain higher levels of exercise intensity, reducing the work of breathing and beneficially modulate vascular reactivity and exercise tolerance during a single bout high-intensity exercise.

The risk of bias of the included studies in this mini-review was performed using the PEDro Scale Tool (0–10) (49) was demonstrated in Table 1 and highlighted the need for more quality in RCT studies in this field of research. In the quality assessment, we observed that 100% of the studies meet the criteria of between-group comparison, 88% eligibility criteria and point estimates and variability, 77% random allocation and baseline comparability, 44% blinding assessors, adequate follow-up and intention-to-treat analysis, 11% concealed allocation, and 0% meet the criteria of blinding subjects and therapists.

## Conclusion

Patients with coexistence of COPD-CHF present a further impaired exercise capacity, daily life activities and health status. Cardiopulmonary rehabilitation, involving a multifaceted program and professionals prepared and attentive to multi-morbidity, aimed at improving aerobic capacity, respiratory and peripheral muscle strength, and endurance, appear to be safe, and effective in improving symptoms, exercise capacity and functionality. However, further randomized clinical trials deserve to be conducted to investigate in a higher level of quality the exercise-based rehabilitation in COPD-CHF overlap in a more systematically way in terms of criteria of inclusion, types of CHF (preserved or reduced), components and outcomes as exacerbation rates, morbidity, and mortality.

## Author Contributions

RGM, AG-A, DB-D, and SD: conceptualization and writing—original draft. EW, FRC, CLG, and GDB: literature collection and visualization. AB-S: conceptualization, writing—review, and editing. All authors contributed to the article and approved the submitted version.

## Funding

This mini-review was sponsored by the Fundação de Amparo à Pesquisa do Estado de São Paulo, São Paulo, Brazil (FAPESP N° 2015/26501-1 and 2018/03233-0). AB-S is an Established Investigator (Level 1B) of the Conselho Nacional de Desenvolvimento Científico e Tecnológico (CNPq N° 443687/2018-8), Brazil.

## Conflict of Interest

The authors declare that the research was conducted in the absence of any commercial or financial relationships that could be construed as a potential conflict of interest.

## Publisher's Note

All claims expressed in this article are solely those of the authors and do not necessarily represent those of their affiliated organizations, or those of the publisher, the editors and the reviewers. Any product that may be evaluated in this article, or claim that may be made by its manufacturer, is not guaranteed or endorsed by the publisher.
